# A nationwide study of patients operated for cervical degenerative disorders in public and private hospitals

**DOI:** 10.1038/s41598-022-17194-z

**Published:** 2022-07-27

**Authors:** Elisabet Danielsen, Christer Mjåset, Tor Ingebrigtsen, Sasha Gulati, Margreth Grotle, Jan Håkon Rudolfsen, Øystein P. Nygaard, Tore K. Solberg

**Affiliations:** 1 Department of Clinical Medicine, UiT The Arctic University of Norway, Tromsø, Norway; 2 Faculty of Medicine, University of Oslo, Oslo, Norway; 3 Department of Neurosurgery and the Norwegian Registry for Spine Surgery (NORspine), University Hospital of North Norway, Tromsø, Norway; 4 Australian Institute of Health Innovation, Macquarie University, Sydney, Australia; 5 Department of Neurosurgery, St. Olavs Hospital, Trondheim, Norway; 6 Department of Neuromedicine and Movement Science, Norwegian University of Science and Technology, Trondheim, Norway; 7 Centre for Intelligent Musculoskeletal Health, Faculty of Health Science, Oslo Metropolitan University, Oslo, Norway; 8 Communication Unit for Musculoskeletal Disorders (FORMI), Oslo University Hospital, Oslo, Norway; 9 Department of Community Medicine, UiT The Arctic University of Norway, Tromsø, Norway; 10 National Advisory Unit on Spinal Surgery, St. Olavs Hospital, Trondheim, Norway

**Keywords:** Medical research, Spinal cord diseases, Health care, Health services

## Abstract

During the last decades, there has been an increase in the rate of surgery for degenerative disorders of the cervical spine and in the use of supplementary private health insurance. Still, there is limited knowledge about the differences in characteristics of patients operated in public and private hospitals. Therefore, we aimed at comparing sociodemographic-, clinical- and patient management data on patients operated for degenerative cervical radiculopathy and degenerative cervical myelopathy in public and private hospitals in Norway. This was a cross-sectional study on patients in the Norwegian Registry for Spine Surgery operated for degenerative cervical radiculopathy and degenerative cervical myelopathy between January 2012 and December 2020. At admission for surgery, we assessed disability by the following patient reported outcome measures (PROMs): neck disability index (NDI), EuroQol-5D (EQ-5D) and numerical rating scales for neck pain (NRS-NP) and arm pain (NRS-AP). Among 9161 patients, 7344 (80.2%) procedures were performed in public hospitals and 1817 (19.8%) in private hospitals. Mean age was 52.1 years in public hospitals and 49.7 years in private hospitals (*P* < 0.001). More women were operated in public hospitals (47.9%) than in private hospitals (31.6%) (*P* < 0.001). A larger proportion of patients in private hospitals had high education (≥ 4 years of college or university) (42.9% vs 35.6%, *P* < 0.001). Patients in public hospitals had worse disease-specific health problems than those in private hospitals: unadjusted NDI mean difference was 5.2 (95% CI 4.4 – 6.0; *P* < 0.001) and adjusted NDI mean difference was 3.4 (95% CI 2.5 – 4.2; *P* < 0.001), and they also had longer duration of symptoms (*P* < 0.001). Duration of surgery (mean difference 29 minutes, 95% CI 27.1 – 30.7; *P* < 0.001) and length of hospital stay (mean difference 2 days, 95% CI 2.3 – 2.4; *P* < 0.001) were longer in public hospitals. In conclusion, patients operated for degenerative cervical spine in private hospitals were healthier, younger, better educated and more often men. They also had less and shorter duration of symptoms and seemed to be managed more efficiently. Our findings indicate that access to cervical spine surgery in private hospitals could be skewed in favour of patients with higher socioeconomic status.

## Introduction

Degenerative spine disorders are a leading cause of lost disability-adjusted life years, work absence worldwide, and place an economic burden on the whole society ranging from individuals, industry and governments^1,2^. Degenerative changes in the cervical spine increase with age^3^, and more than 80% of people above the age of 50 have spondylotic changes on magnetic resonance imaging (MRI)^4^. Although degenerative changes in the cervical spine are commonly encountered on MRI, they do not necessarily correlate well with the presence or severity of symptoms. Characteristic spondylotic changes are disc herniation, osteophyte formation, ligament hypertrophy and ossification, and can lead to narrowing of the spinal canal and/or nerve root foramen. This can lead to mechanical compression of the nerve root(s) (degenerative cervical radiculopathy (DCR)) or spinal cord (degenerative cervical myelopathy (DCM)), or both. Often, conservative treatment can provide sufficient symptom relief. The indication for surgical treatment depends on severity and progression of neurological symptoms^3,5^. According to a study from the Norwegian Registry for Spine Surgery (NORspine), surgery for DCR increased by 86.5% between 2008 and 2014^6^. A similar rise has been seen in Finland^7^ and the United States^8^. Increased availability of MRI diagnostics, an aging population, advances in surgical techniques, and easier access to specialist health care services have been associated with the rise in surgical spine rates^8,9^.

Norway has universal health insurance, with public hospitals being the primary source of specialised care. Like many single payer systems, the Norwegian Health Services are facing challenges with treatment capacity and long waiting times for elective surgery^10,11^. Concurrently, there has been a steady increase in the number of people with supplementary private health insurance in Norway^11^. To ensure equal access to treatments and reduce waiting times, the government has introduced several demand and supply side policies, such as giving patients free choice of hospital, and incentivising the private sector through reimbursement contracts with Regional Health Authorities^11^. More capacity in the private sector may also increase demand which could lead to overuse, especially if the threshold for offering surgical treatment is lowered, particularly in wealthier regions where more people can afford private insurance or to pay directly out-of-pocket. A previous study showed that surgery performed in the private sector did not compensate for the differences in rates of cervical spine surgery in residential areas^6^.

To the best of our knowledge, no previous study has compared patient management data and clinical features of patients operated for DCR and DCM in private and public hospitals. Such information would be valuable for administrators and policymakers, specifically for capacity planning and ensuring equal access to health services. The aim of this population-based study was to compare sociodemographic-, clinical- and patient management data on patients operated for DCR and DCM in public and private clinics in Norway.

## Materials and methods

### Study design and setting

Cross-sectional, population-based study including 9161 patients operated for DCR and DCM at 13 public and private hospitals (specialist health care) in Norway. This paper is consistent with Strengthening the Reporting of Observational Studies in Epidemiology (STROBE) statement^12^.

### Data source

Data were obtained from the NORspine – a government funded registry intended for quality control and research. Currently (2020) the NORspine comprises all hospitals performing cervical surgery (100% coverage), recording 82% of the cases (completeness) operated in Norway^13^.

### Eligibility criteria

Inclusion criteria were consecutive NORspine cases undergoing surgery for DCR or DCM between January 2012 and December 2020. Exclusion criteria were patients with non-degenerative conditions (cancer, primary infections or fracture) and those under the age of 16.

### Measurements

At admission for surgery, patients completed self-administered questionnaires about sociodemographic- and lifestyle factors and patient reported outcome measures (PROMs). Variables included were age, gender, mother tongue, obesity (Body Mass Index (BMI) ≥ 30), smoking, educational level, work status, paresis, duration and severity of symptoms (pain and disability).

At the day of surgery, the operating surgeon recorded information about comorbidity (the American Society of Anesthesiologists Grade (ASA, I-V)), diagnosis, previous cervical surgery, type of surgery, number of levels operated, perioperative complications, duration of surgery (minutes) and hospital stay (days) on a separate form.

### Patient reported outcome measures (PROMs)

The NORspine uses the following PROMs for cervical surgery:

1. Neck disability index (NDI)^14,15^ is a measure of neck pain related disability, consisting of the following items: pain, personal care, lifting, reading, headaches, concentration, work, driving, sleeping and recreation. The score ranges from 0 (no disability) to 100 (maximum disability). The NDI scores can be divided into five categories: no disability (0 to 9), mild disability (10 to 29), moderate disability (30 to 49), severe disability (50 to 69) and complete disability (70 to 100)^16^.
2. EuroQoL-5D (EQ-5D)^17^ is a preference weighted generic measure of health-related quality of life based on the five dimensions: mobility, self-care, activities of daily life, pain and anxiety/depression. Each dimension has three response alternatives: “no”, “mild to moderate” and “severe problems”, and weighted according to UK tariffs^18^. The total score ranges from − 0.596 to 1 (perfect health). Negative values are considered worse than death.
3. Numeric rating scale for neck pain (NRS-NP) and arm pain (NRS-AP)^19^ are measures of pain severity. The score ranges from 0 (no pain) to 10 (worst conceivable pain).

### Statistical analyses

We performed complete case analysis. Since none of the independent covariates had missing values of ≥ 5%, no imputation was made. Missing data on the dependent variable were not imputed^20^. Differences in means for continuous variables were analysed with one-way ANOVA. Categorical variables were compared using the Chi-square test. Differences in baseline disability (NDI) were also adjusted for covariates previously reported to be associated to the dependent variable using a general linear model^21–24^. The covariates were: ASA > II (yes/no), gender (male/female), obesity (BMI ≥ 30, yes/no), smoking (yes/no), educational level (≥ 4 years of college or university, yes/no), cervical myelopathy (yes/no) and paid sick leave (yes/no). Statistical significance level was defined as *P* < 0.05. All statistical analyses were performed using Statistical Package for the Social Sciences (SPSS, Version 26.0, Armonk, NY: IBM, Corp).

### Ethics declarations

This study was conducted in accordance with the Helsinki Declaration. This study was submitted to the Regional Committee for Medical and Health Research Ethics (REK), which evaluated it to be health services research and study for quality control, thus, not in need of their formal approval (2014/1477/REK Sør-Øst). The Data Inspectorate of Norway approved the NORspine protocol. Informed, written consent was obtained from all patients registered in the NORspine.

## Results

A total of 9161 patients underwent surgery for degenerative disorders of the cervical spine, 7344 (80.2%) in public hospitals and 1817 (19.8%) in private hospitals (Table 1). Mean age was 52.1 years in public hospitals and 49.7 years in private hospitals (*P* < 0.001). More women were operated in public hospitals (47.9%) than in private hospitals (31.6%) (*P* < 0.001). The proportion of patients with high education (≥ 4 years of college or university) was lower in public hospitals (35.6% vs 42.9%; *P* < 0.001). Patients in public hospitals were more often obese (BMI ≥ 30) (24.7% vs 22.0%; *P* < 0.015), had more comorbidity (ASA > II) (11.0% vs 2.9%; *P* < 0.001) and more likely to smoke (31.4% vs 22.2%; *P* < 0.001).

**Table 1:** Sociodemographic and lifestyle characteristics of patients operated for cervical degenerative disorders in public and private hospitals.

	Public Hospitals (N = 7344)	Private Hospitals (N = 1817)	*P* value
Age, years; mean (95% CI)	52.1 (51.9 – 52.4)	49.7 (49.3 – 50.1)	< 0.001
Missing: 0			
Patients > 70 years			< 0.001
Yes, N (%)	411 (5.6)	16 (0.9)	
**Gender**			< 0.001
Female, N (%)	3516 (47.9)	575 (31.6)	
Missing: 0			
**Mother tongue**			0.359
Norwegian, N (%)	6763 (92.2)	1686 (92.8)	
Missing: 6 (0.1)			
**Obese (BMI ≥ 30)**			0.015
Yes, N (%)	1768 (24.7)	396 (22.0)	
Missing: 215 (2.3)			
**Smokers**			< 0.001
Yes, N (%)	2265 (31.4)	398 (22.2)	
Missing: 151 (1.6)			
**ASA**^a^ **ASA grade > II**			< 0.001
Yes, N (%)	785 (11.0)	53 (2.9)	
Missing: 180 (2.0)			
**Higher education (≥ 4 years of college or university)**			< 0.001
Yes, N (%)	2540 (35.6)	767 (42.9)	
Missing: 235 (2.6)			
**Work status; N (%)**			< 0.001
Working full time	1987 (27.1)	914 (50.4)	
Home workers	62 (0.8)	7 (0.4)	
Student	31 (0.4)	1 (0.1)	
Age pension	699 (9.5)	35 (1.9)	
Unemployed	114 (1.6)	8 (0.4)	
Sick leave	2569 (35.0)	750 (41.3)	
Partly sick leave	200 (2.7)	46 (2.5)	
Rehabilitation	520 (7.1)	23 (1.3)	
Disability pension	965 (13.1)	23 (1.3)	
Missing: 93 (1.0)			

^a^American Society of Anesthesiologists (ASA).

### PROMs

Patients in public hospitals had baseline mean PROM scores (95% confidence interval, CI) indicating worse health status than those operated in private hospitals (Table 2): unadjusted NDI (mean difference 5.2, 95% CI 4.4 – 6.0; *P* < 0.001), EQ-5D (mean difference -0.09, 95% CI (-0.11) – (-0.08); *P* < 0.001), NRS-NP (mean difference 0.6, 95% CI 0.4 – 0.7; *P* < 0.001) and NRS-AP (mean difference 0.5, 95% CI 0.3 – 0.6; *P* < 0.001). The adjusted NDI (mean difference 3.4, 95% CI 2.5 – 4.2, *P* < 0.001) was also statistically significant different between the two groups, but smaller. More patients in private hospitals had “no disability” or “mild disability” on the NDI (32.5% vs 22.5%; *P* < 0.001*)*, whereas more patients in public hospitals had “severe disability” or “complete disability” on the NDI (29.4% vs 16.8%; *P* < 0.001). A higher proportion of patients in the public sector reported duration of pain more than one year in both arms (52.5% vs 29.4%; *P* < 0.001) and the neck (63.2% vs 44.7%; *P* < 0.001).

**Table 2:** Pre-operative PROM scores of patients operated for cervical degenerative disorders in public and private hospitals.

	Public Hospitals (N = 7344)	Private Hospitals (N = 1817)	*P* value
NDI^a^ (unadjusted); mean (95% CI)	40.8 (40.4 – 41.1)	35.5 (34.9 – 36.2)	< 0.001
NDI (adjusted); mean (95% CI)	40.3 (39.9 – 40.7)	36.9 (36.1 – 37.6)	< 0.001
Missing: 834 (9.1)			
**NDI; No disability or mild disability**^b^			< 0.001
Yes, N (%)	1485 (22.5)	565 (32.5)	
**NDI; Severe or complete disability**^c^			< 0.001
Yes, N (%)	1938 (29.4)	292 (16.8)	
EQ-5D; mean (95% CI)	0.46 (0.42 – 0.43)	0.52 (0.51 – 0.53)	< 0.001
Missing: 453 (4.9)			
Baseline NRS-NP^d^; mean (95% CI)	6.0 (5.9 – 6.1)	5.4 (5.3 – 5.6)	< 0.001
Missing: 307 (3.4)			
Baseline NRS-AP^e^; mean (95% CI)	6.2 (6.1 – 6.2)	5.7 (5.6 – 5.8)	< 0.001
Missing: 286 (3.1)			
**Duration of arm pain > 1 year**			
Yes, N (%)	3548 (52.5)	494 (29.4)	< 0.001
Missing: 331 (3.6)			
**Duration of neck pain > 1 year**			
Yes, N (%)	4239 (63.2)	749 (44.7)	< 0.001
Missing: 299 (3.3)			
**Paresis**			< 0.001
Yes, N (%)	5685 (77.5)	1227 (67.7)	
Missing: 241 (2.6)			

^a^Neck disability index.

^b^Mild disability = 10 – 29.

^c^Severe disability = 50 – 69.

^d^Numeric rating scales for neck pain.

^e^Numeric rating scales for arm pain.

### Surgical characteristics

A total of 1295 (20.2%) were operated for DCM in public hospitals and 191 (12.1%) in private hospitals (*P* < 0.001) (Table 3). Duration of surgery (mean difference 29 minutes, 95% CI 27.1 – 30.7; *P* < 0.001) and length of hospital stay (mean difference 2 days, 95% CI 2.3 – 2.4; *P* < 0.001) were less in private hospitals than public hospitals. Only 5.7% of all surgeries in public hospitals were day surgeries compared to 97.6% in private hospitals. More patients in public hospitals had been operated on three or more levels (5.0% vs 1.4%, *P* < 0.001). A larger proportion of patients in public hospitals had previously been operated (16.4% vs 11.1%; *P* < 0.001). More perioperative complications were seen in public hospitals (0.8%) than private hospitals (0.2%;* P* < 0.001).

**Table 3:** Surgical characteristics of patients operated for cervical degenerative disorders in public and private hospitals.

	Public Hospitals (N = 7344)	Private Hospitals (N = 1817)	*P* value
Duration of surgery (minutes); mean (95% CI)	85.4 (84.6 – 86.3)	56.6 (44.6 – 57.4)	< 0.001
Missing: 25 (0.3)			
**Day surgery**			
Yes, N (%)	418 (5.7)	1770 (97.6)	< 0,001
Missing: 16 (0.2)			
Days of hospital stay; mean (95% CI)	2.36 (2.32 – 2.40)	0.02 (0.01 – 0.03)	< 0.001
Missing: 141 (1.5)			
**Previously operated on the same level**			< 0.001
Yes, N (%)	434 (5.9)	71 (3.0)	
**Previously operated on a different level**			< 0.003
Yes, N (%)	788 (10.7)	152 (8.4)	
**Previously operated**			< 0.001
Yes, N (%)	1203 (16.4)	201 (11.1)	
Missing: 64 (7)			
**Number of levels operated; N (%)**			< 0.001
1 level	5022 (69.1)	1110 (61.2)	
2 levels	1878 (25.9)	678 (37.4)	
≥ 3 levels	363 (5.0)	26 (1.4)	
Missing: 84 (0.9 )			
**Operated for degenerative cervical myelopathy**			< 0.001
Yes, N (%)	1295 (20.2)	191 (12.1)	
Missing: 1157 (12.6)			
**Type of surgery; N (%)**^a^			< 0.001
ACDF^b^			
Cervical disc herniation	4181 (56.9)	1180 (64.9)	
Decompression for spondylosis without disc herniation	1639 (22.3)	313 (17.2)	
Cage	5615 (96.5)	1467 (98.3)	
Bone graft	4 (0.1)	0 (0.0)	
Additional stabilisation with anterior plate	165 (2.8)	17 (1.1)	
ACDA^c^	67 (1.2)	2 (0.1)	
Posterior cervical fusion	54 (0.7)	1 (0.1)	
Posterior decompression	1212 (16.5)	292 (16.1)	
Other	258 (3.5)	31 (1.7)	
Missing: 0			
**Complications; N (%)**			< 0.001
Dural tears	25 (0.4)	1 (0.1)	
Nerve root injury	11 (0.2)	0 (0.0)	
Other nerve injury	4 (0.1)	0 (0.0)	
Wrong level surgery	2 (< 0.0)	0 (0.0)	
Misplacement of implant	0 (0.0)	0 (0.0)	
Bleeding	2 (< 0.0)	0 (0.0)	
Respiratory complications	2 (< 0.0)	0 (0.0)	
Anaphylactic reaction	1 (< 0.0)	0 (0.0)	
Medulla injury	2 (< 0.0)	0 (0.0)	
Esophageal injury	3 (< 0.0)	1 (0.1)	
Cardiovascular complications	2 (< 0.0)	0 (0.0)	
Large blood vessel injury	4 (0.1)	0 (0.0)	
Missing: 0			

^a^Some patients can have more than one type of surgery.

^b^Anterior cervical discectomy and fusion.

^c^Anterior cervical disc arthroplasty.

## Discussion

In this Norwegian population-based study, we found that patients operated for cervical degenerative spine disorders in private hospitals were younger, more often men, better educated, healthier and were less likely to smoke and be obese. These characteristics have been associated with a higher socioeconomic status, suggesting that access to private health services may be skewed ^25–27^. In addition, the differences in baseline characteristics have been linked to a better prognosis, i.e., better surgical outcomes ^28–30^. Studies comparing the effectiveness of surgery performed in public and private hospitals should therefore be adjusted for these factors.

The effect sizes of differences in preoperative PROM scores were consistent and statistically significant, but relatively small (NDI = 40.8 vs 35.5, NRS-NP = 6.0 vs 5.4, NRS-AP = 6.2 vs 5.7), i.e. clearly lower than the minimally clinical important difference (NDI = 7.5 points, NRS-NP and NRS-AP = 2.5 points)^31^. Both sectors operated patients with a mean NDI score corresponding to moderate pain related disability. However, the proportion of patients with mild disability was 10.0% higher, and the proportion of patients with severe disability was 12.6% lower in private hospitals. The differences in NDI score also remained statistically significant when adjusting for sociodemographic features, comorbidity and working capability. Accordingly, we think it is justified to state that patients operated in the private sector had less disease-specific health problems.

Patients operated in private hospitals reported shorter duration of neck and arm pain, indicating better access to surgical treatment despite less severe health problems. This could indicate unwarranted and skewed access to cervical spine surgery related to socioeconomic segregation. One way to evaluate variations in utilisation and access to treatments is through Wennberg’s framework^32^. Most of the surgery for degenerative cervical spine can be categorised as preference- and supply-sensitive care. This means that the indication for surgery is relative, different treatment options exist and the incidence rate of surgery in a population is dependent on treatment capacity and preferences. In preference and supplier sensitive systems, health disparities and overuse of treatments may occur ^32^. Information concerning unwarranted variation would be valuable to policy makers in single payer systems. To evaluate treatment effectiveness and if there is overuse of cervical spine surgery, costs and surgical outcomes need to be taken into account in future studies.

The lower complication rate and the shorter duration of surgery and hospitalisation we found among patients managed in the private sector are in line with previous reports ^33,34^, and could in part be explained by the differences in patient populations. Another explanation may be that public hospitals are mandated to train residents, while only specialists perform operations in private clinics. Surgeons and operation teams in private clinics also tend to be more subspecialised, and in Norway they do not have to handle emergency cases. Patients with less severe health problems could be managed efficiently by private hospitals. Competitive tendering of health services can therefore give positive social welfare effects through reduced waiting times and higher efficiency ^35^.

### Strengths and limitations

The main strength of this study is the comprehensive population-based data set ensuring high external validity. However, a selection bias could be present if patients in the NORspine differ from those who are not included (18%). Emergency care is the main cause of why patients are not included in the registry ^13^. Misclassification of diagnoses or procedures can potentially lead to information bias ^36^. Another limitation is the lack of data on patients’ income, ethnicity and exact waiting time before surgery. This could provide more information about social disparity and differences in performance between public and private hospitals.

## Conclusion

Patients operated for degenerative cervical spine disorders in private hospitals were healthier, had shorter duration of symptoms and seemed to be managed more efficiently than those treated in public hospitals. The access to cervical spine surgery in private hospitals could be skewed in favour of patients with higher socioeconomic status.

## Data Availability

Available upon request to the corresponding author.

## Abbreviations

MRI: Magnetic Resonance Imaging
DCR: Degenerative Cervical Radiculopathy
DCM: Degenerative Cervical Myelopathy
NORspine: The Norwegian Registry for Spine Surgery
STROBE: Strengthening the Reporting of Observational Studies in Epidemiology
PROMs: Patient Reported Outcome Measures
BMI: Body Mass Index
ASA: American Society of Anesthesiologists
NDI: Neck Disability Index
EQ-5D: EuroQoL-5D
NRS: Numeric Rating Scale
